# Books and Authors

**Published:** 1908-03

**Authors:** 


### BOOKS AND AUTHORS.

**Progresive Medicine Vol. IV., December, 1907.** A Quarterly Digest of Advances, Discoveries and Improvements in the Medical and Surgical Sciences. Edited by **Hobart Amory Hare,** M.D., Professor of Therapeutics and Materia Medica in the Jefferson Medical College of Philadelphia. Octavo, 336 pages, with 30 engravings. Per annum in four cloth-bound volumes, $9.00; in paper binding, $6.00, carriage paid to any address. Lea Brothers & Co., Publishers, Philadelphia and New York.

This number is full of good selections, among which we mention a few. Dr. Steele's monograph on diseases of the digestive tract and allied organs brings the subject up to the present time, giving to the student, as well as practitioner, a complete, clear epitome of all that has been added in recent times to our knowledge of normal and faulty digestion, and the means suggested for the alleviation of the latter condition. Dr. John Rose Bradford's chapter on diseases of the kidneys comprises such subjects as hydronephrosis, renal tumors in children, arteriosclerosis of the renal arteries, renal hematuria and hydremia. Perhaps the subjects of most practical interest are arteriosclerosis and hypertension. The reader will find the article full of interest and value.

Dr. Joseph C. Bloodgood contributes the article on surgery of the extremities, fractures, dislocations, tumors, surgery of the joints, shock, anesthesia and infections. Under anesthesia are summarised the extremely valuable experiments of Crile in the resuscitation of animals apparently dead from chloroform, ether or asphyxia, by artificial infusion with adrenalin and saline solution, together with Crile's conclusions. Bier's induced hyperemia in chronic and acute infections is described, and scopolamine, morphia narcosis, spinal anesthesia and local anesthesia, with their comparative results, will be found in this chapter, which is profuesly and excellently illustrated.

Dr. William T. Belfield contributes the article on genitourinary diseases, which will be found to contain a quantity of new material as well as a careful study of the prostate and the comparative value of the different operations proposed for its relief when diseased. Here also is a rough study of the sequelæ of gonorrhea. Dr. H. R. M. Landis's practical therapeutic referendum gives new things that have developed in the field of therapeutics. Here there will be no disappointment, for Dr. Landis has the gift of choosing the good and refusing the useless. As we have remarked often before, this is one of the best condensations of the literature of the period that is issued by the medical press.

---

**A Handbook of Cutaneous Therapeutics.** By **W. A. Hardaway,** A.M., M.D., Professor of Diseases of the Skin and Syphilis, and **Joseph Grindon,** Ph.B., M.D., Professor of Clinical Dermatology and Syphilis in Washington University, St. Louis. 12mo, 606 pages. Lea Brothers & Co., Philadelphia and New York, 1907. (Cloth, $2.75, net.)

Though this is the first edition of this book in its present form, it is yet based upon Hardaway's manual of skin diseases which is well known to the medical profession. In this handbook, Hardaway has given the description of the various diseases and dealt with their treatment, while Grindon has prepared the special sections on radiotherapy, the high-frequency current, galvanism, faradism and minor surgical procedures. Isadore Dyer has permitted the authors to use his experience, which has been large, concerning the treatment of leprosy, while Good, of Saint Louis, has contributed useful points relating to the preparation of ointments.

Skin diseases present in general the most perplexing problems for the general practitioner, hence it has been the aim of these authors to smoothe the pathway as much as possible that leads to their correct solution; in short, it is a book for the physician engaged in everyday practice upon which he can lean for advice. He will not be disappointed, as it clears up many doubtful points, and is a safe guide. It takes its place readily among the better class of handbooks relating to cutaneous affections.

**American Edition of Nothnagel's Practice. Diseases of the Heart.** By **Prof. Th. von Jurgensen,** of Tubingen; **Prof. Dr. L. Krehl,** of Griefswald; and **Prof. Dr. L. von Schrotter,** of Vienna. Edited, with additions, by **George Dock,** M.D., Professor of Medicine, University of Michigan, Ann Arbor. Octavo of 848 pages, illustrated. Philadelphia and London: W. B. Saunders Company, 1908. (Cloth, $5.00; half morocco, $6.00, net prices.)

No more interesting nor important study can engross the attention of the actively practising physician of the present day than diseases of the heart. They are to be investigated from every viewpoint,—clinical, pathological, functional, neurotic, and organic. It is doubtful if ever before so complete a treatise on these diseases has been issued,—certainly never one so large or exhaustive as this.

We hear nowadays so much about "heart failure," a term that is constantly in the mouths of the laity, and often used by physicians without rhyme or reason, that it is important to study this condition carefully with a view to ascertain its precise status. This has been done by Jurgensen and he has expressed his views of the subject which he records in this book and which constitutes the opening chapter, under the head of insufficiency or weakness of the heart. It will well repay reading by both physician and surgeon; especially should the latter be able to determine for himself as to the ability of the heart to withstand the shock of anesthetic and operation.

In a section of nearly three hundred and fifty pages Krehl, Griefswald, deals with diseases of the myocardium and nervous diseases of the heart. This is a most important section and we have not seen the subjects handled so well in any other treatise. The large group of affections falling under this head makes it very essential for every physician to familiarise himself with its teachings. We commend the entire treatise for its excellence and scientific treatment of diseases of the heart.

---

**Modern Clinical Medicine.** Vol. IV. Diseases of the Nervous System. Edited by **Archibald Church,** M.D., Professor of Nervous and Mental Diseases and Medical Jurisprudence in the Northwestern University Medical Department, Chicago. Authorised translation from "Die Deutsche Klinik" under the general editorial supervision of Julius L. Salinger, M.D. Octavo, pp. 1226. Illustrated. New York and London: D. Appleton & Co. (Cloth, $7.00.)

This remarkable series of German "clinics" is comprised in four volumes, three of which already have been considered in the JOURNAL. It is appropriate that the final volume, now presented should deal with diseases of the nervous system, and that so distinguished a specialist in that department of medicine as Dr. Church should appear as its editor. He may feel complimented, however, to assume editorial control of a work, the text of which is made up by such eminent contributors. Church, himself, is a recognised authority in this field, his treatise written in conjunction with Peterson being one of the accepted textbooks by the better colleges.

The section on general neurological diagnosis by Schuster, Berlin, is full of interest and would well repay careful reading by every physician as it contains much information that the general practitioner should possess. The section on lumbar puncture performed for diagnostic or therapeutic purposes by Quincke, Kiel, is exceedingly instructive, being the best exposition of the subject we have read, and is amply illustrated. One of the sections to which we invite special attention is that on tabes dorsalis by Erb, Heidelberg, in which the latest thought on locomotor ataxia is set forth, and syphilis as a casual factor is discussed in detail. Progressive muscular atrophy is another topic of importance, one that is attracting a good deal of attention, and one that invites careful study. It is dealt with exhaustively by Schultze, of Bonn, and his articles merit unstinted praise.

The paralyses are all handled in a most satisfactory manner and considerable additional light is thrown upon them by the several authors who write concerning the topic. It would be a great undertaking to prepare an exhaustive review treating section by section with analytical precision and it would, also, be quite unnecessary in these busy days to ask our readers to wade through so much material. The better way would be for each one to obtain the book for himself and accord to it a thorough reading. It will well repay the expenditure of the time and money such a plan would entail.

**A Manual of Hygiene and Sanitation.** By **Seneca Egbert,** M.D., Professor of Hygiene in the Medico-Chirurgical College, Philadelphia. Fourth edition. 12mo, 498 pages, with 93 illustrations. Lea Brothers & Co., Philadelphia and New York, 1907. (Cloth, $2.25, net.)

As the years advance more and more attention is being paid to the subjects dealt with in this book. Preventive medicine is the shibboleth of the educated physician of the period and the topics discussed in this book,—hygiene and sanitation,—lie at the very foundation of the subject. When this book first appeared it received favorable mention in the JOURNAL, as have the subsequent editions. Now, since it has been revised in detail, some of it rewritten, and all reviewed we can scarcely recall its equal, certainly not its superior as a manual on the topics of which it treats. In this edition the relation of opsonins to immunity is discussed, which will meet the wishes of the yearner for novelty; while the author has delved into the vital statistics of the census bureau, and of some of the larger cities to obtain data of value bearing on hygiene. It would be a good plan for municipal and town boards of health throughout the country to adopt this manual as a guide.

---

**A Textbook of Physiological Chemistry.** By **Charles E. Simon,** M.D., Professor of Clinical Pathology in the Baltimore Medical College. Third edition. Octavo, 490 pages. Lea Brothers & Co., Philadelphia and New York, 1907. (Cloth, $3.25, net.)

The subject matter of this book is presented in a fashion to meet the trend of medical college teaching. The requirements of the present day are a knowledge of general chemistry for entrance, and then a limitation of its study to the relation chemistry bears toward physiology and pathology. Professor Simon has created a work that is both a textbook for the lecture room and a guide for work in the physiologic and chemical laboratory.

The present edition witnesses the bringing forward of the book to the present date without increasing its size or materially changing its appearance. It deals with that branch of chemistry which concerns the medical student in particular, for presumably he has acquired a knowledge of general chemistry during his academic training. Simon has arranged his book with that end in view and made it, therefore, the most practical textbook of its kind. The albumins, the carbohydrates, the fats, and the ferments are presented in the clearest manner possible, while the chapters on the urine and the blood are exceptionally well written, besides being expressive of the latest scientific thought.

The claims of this work upon the physician in active practice must not be overlooked, because for him it is far and away the most helpful book he can place in his library, dealing as it does with questions relating both to the prevention of disease and the restoration to health of those who are ill. These questions lie at the very threshold of medicine and must not be neglected.

---

**A Manual of the Practice of Medicine.** By **A. A. Stevens, A.M., M.D.,** Professor of Therapeutics and Clinical Medicine in the Woman's Medical College of Pennsylvania. Eighth Edition. 12mo of 558 pages, illustrated. Philadelphia and London: W. B. Saunders Company, 1907. (Fleible leather, $2.50 net.)

Probably no work of this class,—no manual of the practice of medicine,—has been reprinted and issued in as many revised editions as this one. This fact gives token both of its popularity and genuine worth. It contains the essential points of the practice of medicine arranged in such a manner as to be easily accessible for quick reference. The type, too, is of the proper size to make the text agreeable to the eye, while the subheads and sideheads in heavy faced type make the topics and subtopics readily discernable. Bound in flexible covers of black leather, with rounded corners and red edges, it is one of the handsomest volumes of its size that comes to our table.

To make the eighth edition a strictly modern book the author has revised the entire text, as well as rewritten some of it, while new material has been added, to freshen it wherever needed. We cannot speak too highly of this manual as a coach for the student and a reference book for the practitioner.

---

**Heart Disease and Blood Pressure.** By **Louis Faugeres Bishop,** A.M., M.D., Clinical Professor of Heart and Circulatory Diseases, Fordham, University, New York. Second edition. 12mo. pp. 120. New York: E. B. Treat & Company. ($1.00.)

The strenuous life of the workers and worriers of the present day makes serious demand upon the energising forces of the human economy. The heart in particular undergoes great strain and often is the medium through which nerve tire first manifests itself. At all events a tired heart frequently occasions more discomfort than one with real disease.

In this essay which is unusually well written, Bishop has described the various conditions of bloodpressure whether depending upon organic changes or upon vasomotor spasm. It is a very readable and instructive little volume. This edition includes the observations incident to nearly four years additional experience, beyond that recorded in the first edition. The author also has advanced some interesting theories bearing on some phases of the subject, and has slightly changed the title of the brochure. We advise all interested to procure the book and read it with care. It will prove entertaining as well as instructive.

---

**The Commoner Diseases of the Eye.** By **Casey A. Wood,** M.D., Professor of Ophthalmology, Northwestern University, Chicago, and **Thomas A. Woodruff,** M.D., Ophthalmic Surgeon, St. Luke's Hospital, Chicago. 12mo, pp. 598. Illustrated. Third edition. Chicago: W. T. Keener & Co. (Cloth, $2.50.)

Ophthalmologists have recognised this book as among the better smaller treatises on the eye, although it is intended primarily for students and for physicians in general practice. It makes plain the diagnosis and treatment of many diseases of the eye, which the authors are pleased to designate as affections of the visual tract. The commoner diseases of the eye are admirably set forth and especially are the methods of examination described in a clear fashion. Many injuries of the eye and its appendages are described and directions given for their repair or treatment. The nasal cavities and the sinuses as they may affect the eye are dealt with in a most instructive way, while the illustrations follow the text and elaborate it with clearness. Finally, the make-up of the book is most satisfactory. We are unfamiliar with a better book of its class and we think the authors have rendered a distinct service to the profession in this most excellent manual.

**The Pancreas.** Its Surgery and Pathology. By **A. W. Mayo Robson,** D. Sc. (Leeds) F. R. C. S. (Eng.) London, and P. J. Cammidge, M.D. (Lond.) D.P.H. (Camb.) Octavo, pp. 546. Illustrated. Philadelphia and London: W. B. Saunders Co. (Price, $5.00.)

The senior author of this work has been recognised for a long time as authority on the surgery of the pancreas; indeed, it may be said that he has played a principal part in rescuing that organ from the comparative obscurity that hovered over it for so long a time. Robson is one of that small but enlightened group of surgeons who believes that most of the diseases of the pancreas are recognisable during life, and he, himself, has contributed in no small degree to improve the methods of diagnosis whereby most of the affections of this organ can be detected, thus making those conditions that come within the domain of surgery clearer, separating the curable and incurable by sharper lines.

The pathology of the pancreas is well brought out by Robson, and the chapter dealing with this topic is one of the most interesting in the book. It makes clear much that has been obscure heretofore; and the next one, on fat necrosis, is of absorbing interest. Diabetes is necessarily of great interest to the clinician, hence the chapter on that phase of the subject appeals to him. Injuries of the pancreas will attract the attention of the surgeon at once and he will find this chapter of great value.

The illustrations, though few in number, are clear and lend an increase of interest to the book. Opie is naturally the most quoted American author in the treatise, he having made more original investigations relating to the pancreas than any other American.

The entire treatise constitutes a contribution to the literature of medicine that is second to none for the year, and should command the attentive reading of everyone interested in the subject.

---

**International Clinics.** A Quarterly of Illustrated Clinical Lectures and especially prepared articles on Treatment, Medicine, Surgery, Neurology, Pediatrics, Obstetrics, Gynecology, Orthopedics, Pathology, Dermatology, Ophthalmology, Otology, Rhinology, Laryngology, Hygiene and other topics of interest to students and practitioners. Edited by **W. T. Longcope,** M.D., Volume IV., seventeenth series. Philadelphia and London: J. B. Lippincott Co. 1907. (Cloth, $2.00.)

The five articles on treatment in this volume are contributed respectively by Henry, Warthin, Francine, Weil, and Chantemesse. On medicine there are six contributions by Hewlett, McPhedran, Pancoast, Robinson, Swan, and Calmette, respectively. In the department of surgery are six articles contributed respectively by Le Conte, Sights, Cumston, McConnell, Mitchell, and Jopson.

In the department of gynecology are four contributions made respectively by Beyea, Green and Hunter, Wainwright, and Palmer. The two articles relating to genitourinary diseases are presented by Corner and Ballenger respectively; three on orthopedics are by Johnston, Tubby, and Newlin, respectively; three on neurology are by Walton, Stewart, and Gordon respectively; while one on otology is by Lermoyez.

Perhaps we should not make invidious distinction in the presence of authors so distinguished and material so valuable, but we cannot resist the temptation of calling special attention to the paper by Albert Philipe Francine on the etiology and treatment of hemoptosis in pulmonary tuberculosis; to that by Albion Walter Hewlett on the common cardiac arrhythmias; to the one on fractures of the femur by H. P. Sights; to the one on the management of pregnancy, by John W. Wainwright; and to the one on chronic joint disease in children, by W. B. Johnston. While all are excellent these are of great practical utility in everyday practice.

**Five Hundred Surgical Suggestions.** By **Walter M. Brickner,** B.S., M.D., Chief of Surgical Department, Mount Sinai Hospital Dispensary, New York, and **Eli Moschcowitz,** A.B., M.D. Second Series. Duodecimo, 125 pages. New York: Surgery Publishing Co., 1907. (Price, $1.00.)

This small book contains a vast amount of information, most of which is of a very practical kind. It will be found particularly useful to the surgical assistant or junior practitioner. This second edition contains nearly double the number of "suggestions" that were published in the first series. It is a handsome book and is well worth the price at which it is offered.

